# Literary Notes

**Published:** 1901-06

**Authors:** 


### LITERARY NOTES.

The Grosvenor Public Library has added to its medical department the library of the late Dr. F. W. Abbott, which is largely composed of works on the eye and ear. The library is fast developing its medical branch by additions of new books as fast as they appear.

Love's *Medical Mirror*, formerly published at St. Louis, will hereafter issue from New York. Its editorial office is at 537 Fifth Avenue, and its business office at 100 William Street.

Polk's *Medical Register* is in preparation for its 16th year. It embraces names of over 130,000 physicians, with college of graduation, list of colleges, societies, boards of health, journals, mineral springs, hospitals, sanitariums, asylums and other medical institutions; also medical laws of each state. It is indispensable as a book of reference; see advertisement on page xxx.

